# Gluten intake and its possible relationship with pain, skin symptoms, and mental health in the Spanish population: an observational study

**DOI:** 10.3389/fnut.2026.1909893

**Published:** 2026-09-16

**Authors:** David Granizo Bermejo, Antonio Molina-López, Diego Domínguez-Balmaseda, Guillermo García-Pérez-de-Sevilla

**Affiliations:** 1 Universidad Europea de Madrid, Faculty of Medicine, Health and Sports, Department of Real Madrid Graduate School, Madrid, Spain; 2 Facultad de Ciencias Biomédicas y de la Salud, Universidad Alfonso X El Sabio (UAX), Madrid, Spain; 3 Universidad Europea de Madrid, Faculty of Medicine, Health and Sports, Department of Physiotherapy, Madrid, Spain

**Keywords:** chronic pain, food frequency questionnaire, gluten intake, mental health, observational study, skin symptoms, sleep quality, Spanish population

## Abstract

**Introduction:**

Gluten is one of the most widely consumed protein complexes in Western diets, and knowing how much of it is habitually eaten is a prerequisite for interpreting the dose–response evidence on gluten-related disorders, for defining the exposure levels used in gluten challenge studies, and for placing the growing trend of voluntary gluten avoidance in context. No such estimate has been published for the Spanish general population.

**Methods:**

We conducted a cross-sectional observational study to estimate mean daily gluten intake and examine its association with stress, depression, anxiety, chronic pain, sleep quality, and skin symptoms in 526 adults (85.0% women; median age 40.50 years; median BMI 22.81 kg/m^2^) free of celiac disease or gluten intolerance, recruited via an online survey. Gluten intake was estimated with an *ad hoc* food frequency questionnaire applying the Osborne method.

**Results:**

Mean intake in this predominantly female sample was 11.14 10.67 g/day (range: 0–82.40 g/day), higher in men (15.46 g/day) than in women (10.38 g/day) and in younger age groups. Participants reporting skin problems (34.98%) consumed significantly more gluten (*p* = 0.022), though the effect size was small (Cohen’s *d* = −0.057). No significant difference emerged between those with and without chronic pain (*p* = 0.25). Gluten intake showed weak but statistically significant positive correlations with stress (r = 0.140), anxiety (r = 0.100), depression (r = 0.172), and insomnia (r = 0.141).

**Discussion:**

These findings provide the first available estimate of gluten consumption in Spain and suggest a possible role of dietary gluten in extra intestinal symptomatology among non-celiac individuals. They must, however, be read with caution: women accounted for 85.0% of respondents and 57.4% of the sample was aged 35–49 years, so the values reported here describe a predominantly female, middle-aged convenience sample and cannot be taken as a nationally representative reference value for the Spanish population. Intake was also not adjusted for total energy intake, and gluten co-occurs with fructans in the same wheat-based foods, so the weak associations observed cannot be attributed specifically to gluten.

## 1 Introduction

Gluten is a complex mixture of seed storage proteins present in cereals such as wheat (*Triticum* spp.), rye (*Secale cereale*), barley (*Hordeum vulgare*), spelt (*Triticum spelta*), triticale (*x Triticosecale*), and oats (*Avena sativa*) (1). Wheat, rye, and barley belong to the Triticeae tribe and share a set of closely related storage proteins; oats, while taxonomically placed in the Aveneae tribe, are the cereal most phylogenetically proximate to that group. From a biochemical standpoint, gluten proteins fall under the prolamin category, a classification that reflects their notably high content of glutamine and proline residues. These prolamins serve as the principal nitrogen reserve of the seed endosperm.

Clinically, gluten-related disorders are often confused with irritable bowel syndrome, since both conditions share hallmark symptoms abdominal pain, bloating, and changes in bowel habits such as diarrhea or constipation (2, 3). What makes this overlap particularly challenging is that the two conditions can coexist without any shared underlying mechanism. Beyond the gut lumen itself, mounting evidence suggests that the intestinal microbiota may play a meaningful role in the pathogenesis of non-celiac gluten sensitivity (NCGS) (3).

When individuals with NCGS or celiac disease are exposed to gluten, a broad spectrum of digestive symptoms may arise (4–11): osmotic diarrhea or constipation, abdominal distension and pain, spasms, gas, bloating, intestinal hypersensitivity, mucosal inflammation, food intolerances, nausea, vomiting, and a tendency to produce soft, fatty, and malodorous stools. Beyond the intestinal tract, systemic manifestations have also been well-documented (6, 11), including spontaneous abortions, aphthous ulcers, menstrual irregularities, fatigue, seizures, dermatitis herpetiformis, headache, joint and bone pain, peripheral neuropathy, balance disturbances, fertility problems, psychiatric symptoms (depression, anxiety, or even psychosis), delayed menarche, iron-deficiency anemia, and osteoporosis.

A clinical trial by Roncoroni et al. (12) exposed NCGS patients to escalating gluten doses after a gluten-free washout period: low (3.4–4 g/day), moderate (6.7–8 g/day), and high (10–13 g/day). Although the sample was limited and the design was not a randomized controlled trial, the results were informative: lower doses mainly produced mild gastrointestinal complaints, whereas higher doses triggered fatigue and extraintestinal symptoms including headache, joint or muscle pain, skin rashes, and anxiety. Comparable findings have been reported by other groups at doses around 10 g/day (13). Catassi et al. (14) established that as little as 50 mg of daily gluten can produce histological damage (villous atrophy) in celiac patients, and that the safe threshold should remain below 10 mg. Importantly, studies in non-celiac individuals have also shown that 16 g/day can provoke similar symptoms (15), raising questions about gluten tolerance thresholds beyond the celiac spectrum.

Populations following a Western dietary pattern represent the heaviest gluten consumers, given that gluten-containing staples bread, pasta, pastries, and breakfast cereals form the backbone of most European diets. Countries such as Egypt and Iran surpass 180 kg of wheat per person per year (16, 17). In France, celiac disease affects roughly 1% of the population, yet 10.31% of surveyed adults reported actively avoiding gluten (18). The United Kingdom presents a similar picture: celiac disease prevalence stands at approximately 1% (19), while 3.7%–13% of the population avoids gluten or reports sensitivity to it (20); mean daily intake has been estimated at 9.7 g/day, with men consuming more (11.7 g/day) than women (8.2 g/day) (20). German data place celiac disease prevalence at around 0.9% (21), and a Danish national survey of 1,494 adults reported a mean gluten intake of 10.4 g/day (22).

Spain, by contrast, lacks any published estimate of gluten intake in the general population. Celiac disease prevalence ranges from 1.71% in children to 1.3% in adults (23), figures broadly consistent with global data (24, 25). NCGS is estimated to affect around 6% of Spaniards, and a considerably larger share voluntarily reduces gluten consumption without medical advice, often under the assumption that doing so is inherently healthier (26). This self-imposed restriction warrants attention from a nutritional standpoint, since gluten-free commercial products do not automatically confer a healthier dietary profile.

Against this background, the present study set out to estimate mean gluten intake in the Spanish population and to explore its relationship with nutritional knowledge (27), lifestyle variables (physical activity and diet quality), and symptoms commonly associated with gluten sensitivity specifically stress, depression, anxiety, chronic pain, sleep quality, and skin symptoms.

## 2 Materials and methods

### 2.1 Study design and ethical considerations

We conducted a cross-sectional observational study through an online survey to estimate mean daily gluten intake in a sample of the Spanish adult population. The study followed the STROBE reporting guidelines and was carried out in accordance with the ethical principles set out in the Declaration of Helsinki. Prior to participation, all respondents provided written informed consent. Data were collected by a trained evaluator, stored in coded form in a dedicated file, and kept accessible only to the principal investigator.

Regarding personal data management, the informed consent documents stated in accordance with Organic Law 15/1999 of 13 December on personal data protection that participants retained the right to access, rectify, object to, and cancel their data by contacting the principal investigator directly.

### 2.2 Study population

Eligible participants were adults aged 18–75 years. Individuals with a confirmed diagnosis of celiac disease or gluten intolerance were excluded from the study. Because the study was conducted entirely online, eligibility was established exclusively through self-report: participants were asked whether they had received a medical diagnosis of celiac disease or of gluten intolerance, and those answering affirmatively were excluded. No serological, genetic, or histological confirmation was obtained, and no participant was tested for non-celiac gluten sensitivity, wheat allergy, or fructan (FODMAP) intolerance; the term “gluten intolerance” therefore reflects the respondent’s own report and was not further characterized. In addition, irritable bowel syndrome, wheat allergy, and other food allergies were not applied as exclusion criteria. This is relevant on three counts: irritable bowel syndrome shares most of its symptom profile with non-celiac gluten sensitivity; patients with either condition are frequently intolerant to fructans, the most prevalent FODMAP intolerance in Western populations consuming wheat daily; and wheat-based foods contain further immunologically active proteins, such as amylase/trypsin inhibitors, so that reported skin manifestations may represent responses to wheat components other than gluten, or to other constituents of composite products such as pizza. The consequences of these decisions for the interpretation of our findings are addressed in the Limitations section.

### 2.3 Sample size calculation

Sample size was determined to ensure adequate representation of the Spanish population, following the procedure described by Hoppe et al. (22). The calculation used the standard deviation observed in that reference population (σ = 4.4 g/day), a 95% confidence level, a conservative design effect (DEFF = 1.2), and an estimated response rate of 53%. Sampling was stratified by sex and three age bands (18–30, 31–45, and 46–75 years), yielding six analytical strata. The target was 1,500 valid responses (approximately 250 per stratum), which required roughly 472 invitations per stratum (total estimated invitations: 2,832). This procedure produced an approximate margin of error of ±0.60 g/day within each stratum.

### 2.4 Variables

The following descriptive and anthropometric variables were recorded: sex, age, country of origin, country of residence, height (cm), and weight (kg). BMI was subsequently derived from these measurements (kg/m^2^).

#### 2.4.1 Dietary characteristics and gluten intake

Mean daily gluten intake was estimated using a food frequency questionnaire (FFQ) covering the three months preceding data collection, designed ad hoc for this study. The FFQ included 14 gluten containing food items cereals, breakfast cereals, baguette bread, sliced bread, pita bread, toast, tortillas, pasta, pizza, empanada, croissant, muffins, churros, and biscuits selected to reflect habitual dietary patterns in Spain (28).

For each item, participants reported consumption frequency (never; 1–3 times/month; 1–3 times/week; 4–6 times/week; once/day; or more than once/day), portion size (small, medium, or large) using standardized household measures, and the number of units consumed per occasion.

Portion weights were assigned using standard portion size tables and household measure references (29). Foods with high gluten content were classified according to the criteria established by the Federation of Celiac Associations of Spain (FACE).

Gluten intake was derived from the vegetable protein content of the recorded foods, applying the Osborne method (30). Briefly, the total grams consumed of each food were calculated from portion size and reported frequency; the vegetable protein content was then multiplied by a coefficient of 0.8 to obtain an estimate of gluten (31). Protein data were sourced from the Spanish Food Composition Database (BEDCA) and from the nutritional labeling of the included products, as required by the Spanish Agency for Food Safety and Nutrition (AESAN) (32, 33).

#### 2.4.2 Mediterranean diet adherence

Diet quality was assessed with the MEDAS-14 questionnaire, a 14 item tool covering key Mediterranean diet components including olive oil, nuts, fruits and vegetables, fish, and legumes. A total score of 9 or above is taken as indicative of good adherence to the Mediterranean dietary pattern (34).

#### 2.4.3 Nutritional knowledge

Nutritional knowledge was evaluated using a self-administered questionnaire adapted from Sánchez Socarrás et al. (27), consisting of eight questions with four response options each. One point is awarded per correct answer, giving a maximum score of 8. Higher scores on this instrument have been associated with better overall diet quality in several studies (35–37).

#### 2.4.4 Physical activity

Physical activity was measured with version 2 of the Global Physical Activity Questionnaire (GPAQ), a who developed self-report tool that assesses activity during a typical week across three domains: work-related activity, active commuting, and leisure-time exercise. The questionnaire captures both frequency and duration of moderate- and vigorous intensity activities, and results are expressed in MET-minutes per week (38). Physical exercise is relevant to this study because it has been shown to modify intestinal permeability over both the short and long term (39).

#### 2.4.5 Psychological status: stress, depression, and anxiety

The Depression, Anxiety and Stress Scale (DASS-21) is a three dimensional self-report instrument designed to measure the severity of depressive, anxiety, and stress symptoms over the preceding week. Each item is rated on a four point Likert scale ranging from 0 (did not apply to me at all) to 3 (applied to me very much or most of the time). Each of the three subscales contains seven items; subscale scores are obtained by summing the relevant items and multiplying by two, with higher values indicating greater symptom severity. The instrument provides established cut off points for classifying symptom levels as normal, mild, moderate, severe, or extremely severe (40).

#### 2.4.6 Chronic pain

Participants were asked whether they experienced chronic pain defined as pain persisting for three months or longer (41) in anybody region. Those who answered affirmatively were asked to describe its characteristics: anatomical location, frequency (days per month), intensity on a 0–10 scale, and typical episode duration (hours). This variable was included because certain studies have reported a link between chronic pain and increased intestinal permeability (42).

#### 2.4.7 Sleep quality

Sleep quality was assessed using the validated Spanish version of the Athens Insomnia Scale, an eight item questionnaire covering sleep onset, nocturnal and early morning awakenings, total sleep duration and perceived quality, frequency and duration of complaints, subjective distress related to insomnia, and its impact on daytime functioning. Each item is scored from 0 (no problem) to 3 (severe problem), yielding a total score between 0 and 24; a score of 6 or above is considered indicative of a possible insomnia diagnosis (43).

#### 2.4.8 Skin symptoms

Skin manifestations such as eczema, urticaria, dermatitis, and exacerbations of preexisting inflammatory skin conditions have been indirectly linked to increased intestinal permeability and, in certain susceptible individuals, to gluten exposure. The most widely supported hypothesis holds that a disrupted intestinal barrier allows dietary or microbial antigens to enter the bloodstream, triggering systemic immune responses that may manifest at peripheral sites including the skin. In this context, dermal symptoms can serve as a complementary clinical indicator of barrier dysfunction and immune activation potentially related to gluten (44).

### 2.5 Statistical analysis

All statistical analyses were performed using IBM SPSS Statistics version 29.0 for Windows (IBM Corp., Armonk, NY, USA). Variable distribution was first examined with the Kolmogorov-Smirnov test. Descriptive statistics were then calculated: normally distributed variables are reported as mean ± standard deviation, while those departing from normality are expressed as median and interquartile range (IQR). Between-group comparisons used Student’s *t*-test or the Mann-Whitney U test, as appropriate, with effect size quantified using Cohen’s d. Associations between continuous variables were assessed with Pearson’s r or Spearman’s ρ depending on their distribution; where significant correlations were identified, linear regression was performed. All tests were evaluated at a 95% confidence level, with *p* < 0.05 as the threshold for statistical significance.

## 3 Results

### 3.1 Sample characteristics

The final sample comprised 526 participants, 85.0% of whom were women. Median age was 40.50 years (IQR: 13) and median BMI was 22.81 kg/m^2^ (IQR: 4.29).

Mean gluten intake across the whole sample was 11.14 ± 10.67 g/day (range: 0–82.40 g/day).

The median nutritional knowledge score was 7.00 (IQR: 1). Median physical activity reached 520.00 MET-min/week (IQR: 1,240), and median Mediterranean diet adherence (MEDAS) was 9.00 (IQR: 3).

On the DASS-21 subscales, median scores were 6.00 (IQR: 6) for stress, 1.00 (IQR: 2) for anxiety, and 1.00 (IQR: 4) for depression. The median Athens Sleep Questionnaire score was 5.00 (IQR: 5).

### 3.2 Gluten intake by chronic pain status

Chronic pain lasting more than 3 months was reported by 42.21% of the sample. Mean gluten intake was 11.58 ± 10.98 g/day among participants with chronic pain and 10.82 ± 10.45 g/day among those without. The difference between groups did not reach statistical significance (*p* = 0.25).

### 3.3 Gluten intake by skin problem status

Skin problems such as eczema or dermatitis were reported by 34.98% of participants. Those affected consumed a mean of 11.54 ± 11.02 g/day of gluten, compared with 10.93 ± 10.49 g/day among those without skin symptoms. This difference was statistically significant (*p* = 0.022); however, the effect size was small (Cohen’s *d* = −0.057), and the absolute difference between groups amounted to only 0.61 g/day.

### 3.4 Descriptive data by sex and age group

Men consumed substantially more gluten than women (15.46 ± 12.54 g/day vs. 10.38 ± 10.13 g/day). Intake also varied by age group, as shown in Table 1.

**TABLE 1:** Gluten intake by age group (g/day).

Group	Mean	Standard deviation	Minimum	Maximum	*N*
20–34 years	12.10	9.44	0	39.81	137
35–49 years	11.24	11.39	0	82.39	302
≥50 years	9.27	9.76	0	51.13	87
Total	11.14	10.67	0	82.39	526

As the data in Table 1 illustrates, younger participants tended to consume more gluten than those aged 50 or above.

Regarding the distribution of intake across the sample, the 25th percentile (P25) was 3.63 g/day, the median (P50) was 8.09 g/day, P75 was 15.60 g/day, and P90 reached 24.43 g/day.

### 3.5 Correlations between gluten intake and other variables

Gluten intake showed weak but statistically significant positive correlations with nutritional knowledge (ρ = 0.112), stress (ρ = 0.140), anxiety (ρ = 0.100), depression (ρ = 0.172), and insomnia (ρ = 0.141). These associations are summarized in Table 2.

**TABLE 2:** Spearman correlations between gluten intake and study outcomes.

	Nutritional knowledge	Physical activity	DASS21 stress	DASS21 anxiety	DASS21 depression	Athens Sleep Questionnaire
Gluten intake	0.112*	0.049	0.140**	0.100*	0.172**	0.141**
Nutritional knowledge	–	−0.024	−0.029	−0.103*	−0.028	−0.009
Physical activity	–	–	−0.128**	−0.125**	−0.187**	−0.126**
DASS21 stress	–	–	–	0.584**	0.584**	0.472**
DASS21 anxiety	–	–	–	–	0.542**	0.393**
DASS21 depression	–	–	–	–	–	0.431**

Spearman’s correlation test. *P-value < 0.05. **P-value < 0.01. Bold numbers indicate significant correlations with gluten intake.

## 4 Discussion

This study examined mean gluten intake in 526 participants, 85.0% of whom were women. Median age was 40.50 years and the 35–49 year band was the most represented, comprising 302 participants (57.4% of the sample); respondents were therefore predominantly women in early middle age. They showed a healthy BMI, moderate levels of physical activity, reasonable adherence to the Mediterranean diet, and no diagnosis of celiac disease or gluten intolerance. The data revealed that higher gluten intake was associated with a greater prevalence of skin symptoms and with worse scores on mental health and sleep quality measures.

Participants with chronic pain did not differ significantly from those without in terms of gluten intake (*p* = 0.25). That said, the analysis did not distinguish between pain locations — muscle pain, joint pain, and headache were all grouped together which may have obscured condition specific effects. Other researchers have reported similar null findings in patients with migraine (45) and fibromyalgia (46, 47), whereas a relationship with gluten intake does appear to exist in certain chronic pain conditions, notably rheumatoid arthritis (48). In celiac disease, chronic pain can be driven by pro-inflammatory cytokines that sensitize nociceptive pathways, by nutrient deficiencies (cobalamin, iron, calcium, vitamin D), or by the presence of autoantibodies (anti-tissue transglutaminase, anti-endomysium, and anti-deamidated gliadin peptides) (49–51). Because all participants in the present study were free of a celiac diagnosis, the absence of a gluten–pain relationship is perhaps unsurprising.

Skin symptoms, eczema or dermatitis were reported by roughly one in three participants (34.98%). Gluten intake was significantly higher in this subgroup (*p* = 0.022), though the effect size was small and the absolute difference between groups was only 0.61 g/day. This magnitude requires careful interpretation. Total daily energy intake was not estimated in the present study, and gluten is derived almost entirely from cereal-based staples that are themselves major contributors to total energy intake. A difference of 0.61 g/day corresponds approximately to the gluten contained in a few grams of bread, and it is highly plausible that it simply reflects a proportionally higher overall food and energy consumption in the subgroup reporting skin symptoms rather than any gluten-specific effect. Without adjustment for total energy intake — for example by expressing gluten as a density per 1,000 kcal or by applying the residual method — a statistically significant difference of this size cannot be read as evidence of a specific action of gluten, and attributing these symptoms to gluten remains speculative. The same caveat applies to the correlations reported below for the psychological and sleep variables. It should also be noted that skin problems were identified through a self-administered survey, without dermatological examination or clinical confirmation, which introduces a substantial risk of reporting and misclassification bias. This pattern is broadly consistent with the well stablished link between celiac disease and dermatitis herpetiformis (52, 53) or psoriasis (54), even though none of the participants here had a celiac diagnosis. The proposed biological bridge between gluten and skin involvement centers on dysregulation of immunoglobulin A (54), although the precise mechanisms remain incompletely understood. Given the multifactorial nature of skin conditions, the higher gluten intake observed in participants with skin problems most likely reflects a combination of contributing factors rather than gluten exposure alone. A recent hypothesis paper suggests that cutaneous manifestations may in fact serve as a diagnostic marker for NCGS (55), a possibility worth exploring in future work.

The positive associations between gluten intake and stress, anxiety, and depression are consistent with earlier observational data from celiac populations (56–60). The neuropsychiatric dimension of NCGS is still being mapped, but the picture that is emerging includes extraintestinal manifestations such as brain fog, depression, anxiety, chronic fatigue, and peripheral neuropathies. The mechanism most supported by current evidence involves increased intestinal permeability (61, 62): gliadin peptides translocate into the bloodstream, activate the innate immune system, and elevate circulating pro-inflammatory cytokines (63–65). This systemic inflammatory state can breach the blood-brain barrier and disturb cognitive and affective functioning. Intestinal dysbiosis likely amplifies these effects through the generation of neurotoxic metabolites that dysregulate gut-brain axis signaling (66, 67). It is worth noting, however, that overall levels of stress, anxiety, and depression in this sample were low, so these correlations should be treated with caution and ideally replicated in clinical populations carrying a heavier symptom burden.

Sleep quality scores in this sample hovered right at the Athens Insomnia Scale cut off for insomnia, suggesting a subthreshold but clinically relevant level of sleep disruption. The literature on gluten and sleep remains sparse. One notable reference is a large American health survey of 22,274 individuals, which found that people with celiac disease did not show elevated odds of depression or sleep difficulties compared with controls (68) though that study focused on a diagnosed population, making direct comparisons with our non-celiac sample difficult.

Lifestyle variables showed no meaningful interaction with gluten intake. Neither physical activity levels nor Mediterranean diet adherence correlated with the amount of gluten consumed, which suggests that the decision to reduce or avoid gluten is largely independent of broader healthy lifestyle choices. The positive association between nutritional knowledge and gluten intake though weak may reflect the fact that participants with better dietary literacy did not perceive gluten itself as inherently harmful (35–37).

The wide standard deviation observed across gluten intake data points to substantial heterogeneity within the sample. One portion of the population derives much of its diet from processed flour-based products, while another shows minimal intake, possibly driven by the growing trend of voluntary gluten avoidance for perceived health reasons or undiagnosed sensitivities (26).

Skodje et al. (69) conducted a double blind challenge study comparing the effects of gluten and fructans in 60 patients with self-reported gluten sensitivity. The results were striking: abdominal bloating and other intestinal symptoms were significantly worse after the high-fructan condition than after either the placebo or the high gluten condition. The authors concluded that eliminating gluten from the diet simultaneously removes fructans, and that it may be the fructan fraction not gluten that is primarily responsible for the digestive discomfort many people attribute to gluten (70, 71). This finding deserves consideration when interpreting symptom reports in non-celiac populations.

### 4.1 Limitations

Several limitations of this study merit acknowledgment. The survey took more than 20 min to complete, which hampered recruitment and limits the generalizability of findings to Spain’s full adult population of approximately 45 million. Gluten intake was higher in men (15.46 g/day) than in women (10.38 g/day) and in younger age groups, but formal sex-stratified statistical comparisons were not performed because women accounted for 85% of the sample, rendering the two groups highly unequal. Similarly, the ≥50 years subgroup was considerably smaller than the others, making age-stratified analyses statistically inappropriate. Total daily energy intake was not estimated, which precludes adjustment for overall dietary volume, and means that the between-group differences and the correlations reported here may be driven by differences in total food and energy consumption rather than by gluten specifically; energy-adjusted analyses (nutrient density or residual method) should be regarded as an essential step in future work. Several features of the online recruitment strategy further constrain interpretation. Eligibility was based entirely on self-report, so the absence of celiac disease and of gluten intolerance could not be verified serologically or histologically, and no participant was tested for non-celiac gluten sensitivity, wheat allergy, or fructan intolerance. Irritable bowel syndrome, whose clinical picture largely overlaps with non-celiac gluten sensitivity and which frequently coexists with fructan intolerance, was not an exclusion criterion, so an unknown proportion of respondents may belong to these categories. Wheat allergy and other food allergies were likewise not screened for, and wheat-based products contain additional allergenic proteins such as amylase/trypsin inhibitors, while composite foods combine several potential allergens; reported skin symptoms may therefore reflect immune responses to food components other than gluten. Skin problems, chronic pain, and psychological status were all self-reported without clinical confirmation, and online surveys of this type are additionally exposed to self-selection bias, since respondents with a pre-existing interest in diet and gluten are more likely to take part. As with any cross-sectional design, causal inferences cannot be drawn from these data.

### 4.2 Clinical implications

These findings have some practical relevance for clinical practice. Patients presenting with extraintestinal symptoms skin conditions, anxiety, depression, or sleep disturbances may benefit from a routine assessment of gluten intake as a potentially modifiable contributing factor, even in the absence of a formal celiac or NCGS diagnosis. Although diagnostic awareness and the availability of gluten-free products have both improved, many patients continue to report persistent symptoms and diminished quality of life, often linked to fear of cross-contamination and uncertainty about food safety outside the home. The “gluten-free trend” has introduced considerable confusion into the public discourse; future epidemiological surveys should clearly distinguish between individuals with celiac disease, those with NCGS, and those who voluntarily avoid gluten for lifestyle reasons. On a methodological note, the reference value for mean gluten intake in the Spanish population provided here can serve as a benchmark for future gluten challenge studies and cross-country dietary comparisons.

### 4.3 Future research

Several directions seem worth pursuing. Prospective studies with larger, more balanced samples including a greater proportion of men and older adults would allow more robust sex- and age-stratified analyses. Intervention designs, such as double-blind crossover trials comparing gluten-containing and gluten-free diets in non-celiac individuals with extraintestinal symptoms, would help clarify whether the associations reported here reflect a causal relationship. Mechanistic work exploring intestinal permeability biomarkers (e.g., zonulin, fatty acid-binding protein) alongside symptom assessment could shed light on the pathways connecting gluten intake to skin and neuropsychiatric outcomes. Finally, given the overlap between gluten and fructan content in many foods, future studies should consider separating these dietary components to determine their independent contributions to symptom burden in non-celiac populations.

Regarding future therapeutic approaches, CRISPR/Cas9 technology is currently being explored to reduce the immunogenic gluten content of wheat varieties (72–74), which could eventually allow individuals with mild gluten sensitivity to consume wheat-based products with fewer adverse effects. Enzymatic strategies aimed at degrading immunogenic gluten peptides in the gut represent another promising avenue (75). At the immunological level, antigen-specific immunotherapy trials for celiac disease are underway (76–83), and their outcomes may have broader implications for understanding gluten tolerance thresholds across the sensitivity spectrum.

## 5 Conclusion

In this cross-sectional study of Spanish adults, mean gluten intake showed substantial interindividual variability and was higher in younger participants and in men. While gluten intake was not associated with chronic pain, weak but statistically significant associations were observed with skin symptoms and psychological variables, including stress, anxiety, depression, and sleep quality. However, the magnitude of these associations was small, and their clinical relevance remains uncertain. These associations should not be interpreted as evidence of an independent pathological role of gluten in non-celiac individuals. Gluten and fructans co-occur in the same wheat-based foods, and controlled double-blind challenge studies have identified fructans, rather than gluten, as the principal symptom trigger in people with self-reported gluten sensitivity (69, 70); the weak correlations observed here may therefore be entirely attributable to concurrent fructan intake, to a higher total energy intake for which no adjustment was possible, or to the reporting bias inherent in self-administered symptom questionnaires applied without clinical confirmation. Gluten consumption can thus be regarded, at most, as one of several possible correlates of extraintestinal symptoms in the general population, and causality cannot be inferred. Importantly, gluten intake appeared to be independent of overall lifestyle factors such as physical activity and adherence to the Mediterranean diet, but was weakly associated with nutritional knowledge. Overall, this study provides the first descriptive data on gluten intake in a Spanish adult sample — which, given the demographic imbalance described above, should be read as a preliminary estimate rather than a nationally representative reference value — and highlights the need for well-designed longitudinal and interventional studies to clarify the potential role of gluten in extraintestinal manifestations, particularly in individuals without diagnosed Celiac Disease or Non-Celiac Gluten Sensitivity.

## Data Availability

The raw data supporting the conclusions of this article will be made available by the author, without undue reservation.
